# Obesity and triple‐negative‐breast‐cancer: Is apelin a new key target?

**DOI:** 10.1111/jcmm.15639

**Published:** 2020-07-17

**Authors:** Florian Gourgue, Lionel Mignion, Matthias Van Hul, Natacha Dehaen, Estelle Bastien, Valery Payen, Baptiste Leroy, Nicolas Joudiou, Didier Vertommen, Caroline Bouzin, Nathalie Delzenne, Bernard Gallez, Olivier Feron, Bénédicte F. Jordan, Patrice D. Cani

**Affiliations:** ^1^ Metabolism & Nutrition Research Group Louvain Drug Research Institute WELBIO (Walloon Excellence in Life sciences and BIOtechnology) UCLouvain Université catholique de Louvain Brussels Belgium; ^2^ Biomedical Magnetic Resonance Research Group UCLouvain Louvain Drug Research Institute Université catholique de Louvain Brussels Belgium; ^3^ Pole of Pharmacology and Therapeutics Institut de Recherche Expérimentale et Clinique UCLouvain Université catholique de Louvain Brussels Belgium; ^4^ Pole of Pediatrics Institut de Recherche Expérimentale et Clinique UCLouvain Université catholique de Louvain Brussels Belgium; ^5^ Laboratory of Proteomics and Microbiology MS‐Quanta Platform Research Institute for Biosciences University of Mons Mons Belgium; ^6^ Nuclear and Electron Spin Technologies (NEST) Platform Louvain Drug Research Institute (LDRI) UCLouvain Université catholique de Louvain Brussels Belgium; ^7^ de Duve Institute (DDUV) Université catholique de Louvain (UCLouvain) Brussels Belgium; ^8^ Imaging platform 2IP Institut de Recherche Expérimentale et Clinique (IREC) UCLouvain Université catholique de Louvain Brussels Belgium

**Keywords:** apelin, fat mass, high‐fat, obesity–cancer link, triple‐negative breast cancer

## Abstract

Epidemiological studies have shown that obese subjects have an increased risk of developing triple‐negative breast cancer (TNBC) and an overall reduced survival. However, the relation between obesity and TNBC remains difficult to understand. We hypothesize that apelin, an adipokine whose levels are increased in obesity, could be a major factor contributing to both tumour growth and metastatization in TNBC obese patients. We observed that development of obesity under high‐fat diet in TNBC tumour‐bearing mice significantly increased tumour growth. By showing no effect of high‐fat diet in obesity‐resistant mice, we demonstrated the necessity to develop obesity‐related disorders to increase tumour growth. Apelin mRNA expression was also increased in the subcutaneous adipose tissue and tumours of obese mice. We further highlighted that the reproduction of obesity‐related levels of apelin in lean mice led to an increased TNBC growth and brain metastases formation. Finally, injections of the apelinergic antagonist F13A to obese mice significantly reduced TNBC growth, suggesting that apelinergic system interference could be an interesting therapeutic strategy in the context of obesity and TNBC.

## 1 INTRODUCTION

Breast cancer (BC) is the most prevalent type of cancer and the primary cause of cancer‐related death among women with an estimated 2.1 million diagnosed cases and 627.000 deaths in 2018.^1^ This highly heterogeneous disease comprises multiple subsets of BC characterized by different expression patterns of key receptors and protumoral proteins.^2^, ^3^ Therefore, the different classes of BC vary in terms of prognosis, with the most severe form being the triple‐negative breast cancer (TNBC) that accounts for 10%‐20% of all BC. TNBC is characterized by the lack of oestrogen receptor (ER) and progesterone receptor (PR) expression as well as the absence of human epidermal growth factor receptor 2 (HER‐2) up‐regulation, making them incompatible with current targeted therapies.^4^, ^5^, ^6^ Hence, TNBC patients have higher recurrence rate, increased susceptibility to form brain or lung metastases and have a decreased overall survival.^7^, ^8^ Several risk factors for BC have been identified and include family history of breast cancer,^9^ ethnicity,^10^, ^11^ oestrogen exposure^12^, ^13^ and contraceptive use.^14^ Moreover, it has been recently stated that obesity is another factor increasing the risk of developing BC.^15^, ^16^, ^17^ This relation is most described for obese post‐menopausal women suffering from ER‐positive BC as obesity increases aromatase activity in the adipose tissue after the menopause, leading to local oestrogen production.^18^ High concentration of oestrogen can either induce DNA damages that increase the risk of developing cancer,^19^, ^20^ or promote the proliferation of existing ER‐positive BC cells. Regarding the relation between obesity and TNBC, the situation is more complex and still poorly understood. Several epidemiological studies have shown that obese subjects have an increased risk of developing TNBC,^21^, ^22^, ^23^ larger tumours^24^ and shorter disease‐free and overall survival.^25^ As global obesity rates are continuously increasing and that TNBC cannot benefit from current targeted therapies, there is a need to better understand the implications of obesity on TNBC development and progression. Several obesity‐related disorders have already been studied for their potential role in cancer progression such as hyperinsulinaemia, high circulating levels of IGF‐1, inflammation or alteration of adipokine secretion.^17^, ^26^ Among the latter, the obesity‐related increased levels of leptin have been shown to contribute to tumour progression by stimulating cancer cell proliferation, survival and invasion.^27^, ^28^, ^29^ However, owing to leptin physiological functions, the use of leptin receptor antagonist LPrA2 in vivo increased significantly the bodyweight of obese mice.^30^ Apelin is another adipokine whose levels are increased with obesity.^31^ This adipokine is first expressed as a precursor form of 77 amino acids called preproapelin.^32^ Upon sequential cleavages, this precursor form will generate different mature isoforms as apelin‐36, apelin‐17, apelin‐13 or a pyroglutaminated form of apelin 13 (pyr apelin‐13) that is more stable and therefore the major circulating form of the adipokine.^33^ The apelin receptor (Aplnr) is a class A G protein‐coupled receptor that is expressed in many tissues.^34^ The apelin‐Aplrn interaction is involved in several physiological functions as suggested by the wide expression pattern of both ligand and receptor. Apelin functions comprise blood vessels formation, energy metabolism, fluid homeostasis and blood pressure regulation.^35^, ^36^, ^37^ Recently, several correlation studies^38^, ^39^, ^40^ and one study of artificial apelin overexpression in cancer cells^41^ have highlighted a potential implication of apelin in tumour progression. Strikingly, no study has analysed the impact of the obesity‐related increased levels of apelin on tumour growth. Given that obesity is considered as a risk factor for developing TNBC and that the mechanisms are poorly understood, we explored the hypothesis that obesity‐increased levels of apelin could be a major factor contributing to either tumour growth or metastatization, thereby explaining the poor prognosis in TNBC patients.

## 2 MATERIALS AND METHODS

### 2.1 Animal studies

Animal studies were undertaken in accordance with the Belgian law concerning the protection and welfare of animals and were approved by the UCLouvain ethical committee (agreement reference: UCL/2014/MD/026 and 2017/UCL/MD/005). All investigators performing in vivo studies successfully completed FELASA C training.

### 2.2 Mice

All mice were purchased from Janvier Labs (Saint Berthevin, France) and housed in a controlled environment (22 ± 2°C, 12‐hour daylight cycle, lights off at 6 PM) with free access to food and water. Long‐term high‐fat diet experiments were performed on 7‐week‐old female C57BL/6JRj or Balb/cJRj mice. Mice were fed either a normal diet (10% fat, AIN93Mi; Research Diets, New Brunswick, NJ, USA) or a high‐fat diet (60% fat, D12492i; Research Diets) throughout the whole experiment. Short‐term infusion experiments were performed on 9‐week‐old Balb/cJRj or Balb/c nude mice fed a normal diet (AIN93Mi; Research Diets). Bodyweight was followed once a week for each experiment, and for long‐term HFD experiments, body composition was assessed by using 7.5 MHz time domain‐nuclear magnetic resonance (TD‐NMR) (LF50 Minispec; Bruker, Billerica, MA, USA).

### 2.3 Obesity relevant apelin infusion

Every experiment involving apelin was performed with the pyroglutaminated form of apelin‐13 (pyr apelin‐13) (Bachem, Bubbendorf, Switzerland), which is the main circulating form. Apelin infusion of 0.1 µmol/kg/d in sterile PBS was based on circulating apelin quantification in obese mice (^42^) and published studies analysing the impact of chronic increased apelin on energy metabolism (^43^). Apelin infusions were performed with osmotic mini pumps (model 2006; Alzet, Cupertino, CA, USA) allowing a constant infusion up to 6 weeks. Osmotic pumps were used according to manufacturer's instructions. Briefly, pumps were filled under sterile condition with either apelin or PBS before an activation period of 72 hours in 0.9% saline solution at 37°C. Pumps were next installed subcutaneously under isoflurane anaesthesia.

### 2.4 Treatment of apelinergic antagonist

Apelinergic antagonist F13A was purchased from Bachem. Daily intraperitoneal injection of either F13A (0.5 µg/g) or PBS started on the fifth day of TNBC growth until the end of the experiment.

### 2.5 TNBC cell lines

The TNBC 4T1 cell line was acquired from the American Type Culture Collection (ATTC); these cells are derived from Balb/c mice. E0771 cells were purchased from CH3 BioSystems (Amerhest, NY, USA) and are derived from C57BL/6 mice. The 4T1 Luc‐GFP cell line was obtained from Perkin Elmer (4T1 Red FLuc‐GFP, PerkinElmer, Waltham, MA, USA) and grows only in immunodeficient mice. 4T1 cells and 4T1 Luc‐GFP cells were cultured in RPMI‐1640 Glutamax (Thermo Fisher Scientific, Waltham, MA, USA) supplemented with 10% heat‐inactivated FBS (Thermo Fisher Scientific). The E0771 cells were cultured in RPMI‐1640 Glutamax HEPES (Thermo Fisher Scientific) supplemented with 10% heat‐inactivated FBS. All cells were cultured at 37°C in a humidified atmosphere with 5% CO_2_.

### 2.6 Cell proliferation

Cell proliferation was assayed with a 5‐bromo‐2′‐deoxyuridine (BrdU)‐ELISA‐based kit (Roche, Basel, Switzerland) following the provider's instructions. Cells were seeded in complete medium for 24 hours prior to medium replacement by a medium depleted of FBS for 24 hours. Cells were then treated with 0, 50 or 5000 nmol/L of apelin in FBS‐free medium. After treatment for 24 hours, cells were incubated in the presence of BrdU for 1 hour. The amount of BrdU incorporated in the cells was assessed by measuring the absorbance at 370 nm using a plate reader (SpectraMax M2e, Molecular Devices, San Jose, CA, USA), which allowed the quantification of DNA synthesis in replicating cells.

### 2.7 TNBC xenograft

TNBC cells were injected orthotopically into the fourth left mammary gland in Hank's balanced salt solution (HBSS; Thermo Fisher Scientific). For the 4T1 cell line, 2 x 10^5^ cells were injected and 5 x 10^5^ cells for the E0771 cell lines. For long‐term high‐fat diet experiments, tumours were induced after 5 weeks of either normal diet or high‐fat diet. For short‐term infusion experiments, tumours cells were injected after 2 days of infusion. Tumour growths were followed blinded by calliper measures and interrupted around 600 mm^3^. Tumour xenografts were performed on groups of 5‐22 mice depending on tumour growth: 4T1 homogeneous tumour growth allows smaller groups but the E0771 described heterogeneous development has led to the use of bigger groups. For E0771 TNBC tumours, we considered as successfully grown TNBC any tumour above 100 mm^3^ at the end of experiment.

### 2.8 Tissue sampling

At the end of TNBC growth, mice were anaesthetized with isoflurane, and blood was sampled by cardiac puncture. After exsanguination, mice were killed by cervical dislocation. Subcutaneous adipose tissue is immediately immersed in liquid nitrogen. TNBC tumours were collected, any necrotic area was removed, and tumours were cut into halves: one half was stored in liquid nitrogen for mRNA and protein analysis, and the other was prepared for histological analysis. Frozen tumour pieces for mRNA and protein analysis were ground with a pestle in liquid nitrogen to obtain homogeneous tissue powder.

### 2.9 Western blot analyses

Homogeneous tumour powder was lysed in RIPA buffer (Sigma‐Aldrich, Saint Louis, MO, USA) supplemented with 1% of Halt protease inhibitors and Halt phosphatase inhibitors (ThermoFisher). Equal amounts of proteins were separated by SDS–PAGE and transferred to PVDF membranes. Membranes were incubated overnight at 4°C with antibodies diluted 1:1000 in Tris‐buffered saline Tween‐20 containing 1% bovine serum albumin (APLNR antibody Invitrogen, Carlsad, CA, USA #711101; HSP90 antibody Cell Signaling Technology, Danvers, MA, USA #4875). The revelation was performed using a chemiluminescent substrate (SuperSignal^®^ West Pico ThermoScientific) and LAS 500 (GE Healthcare, Chicago, IL, USA). HSP90 protein was chosen as loading control.

### 2.10 RNA preparation and real‐time qPCR analysis

Total RNA was prepared from tissues using TriPure Isolation Reagent (Roche) according to manufacturer's instructions. Quantification and quality of total RNA were performed by running 1 μL of each sample on a nanophotometer (Implen GmbH, Munich, Germany). The cDNA was prepared with the GoScript Reverse Transcriptase System (Promega, Madison, WI, USA), and real‐time qPCR was performed on QuantStudio 5 Real‐Time PCR System using GoTaq qPCR Master Mix (Promega). RPL19 RNA was chosen as the housekeeping gene.

### 2.11 Immunohistochemistry

Tumours were fixed in 4% paraformaldehyde for 24 hours at room temperature before processing for paraffin embedding. For each tumour, three 5‐µm sections spaced 500 µm apart were submitted to antigen retrieval using citrate buffer. Sections were then incubated in BSA 5% in TBS/Triton 0.05% to block non‐specific binding, then overnight at 4°C with primary antibodies for Pecam1 (Cell Signaling Technology). Envision anti‐rabbit secondary polymer antibody was used (Dako‐Agilent, Santa Clara, CA, USA). Stained slides were then digitalized using a SCN400 slide scanner (Leica Biosystems, Wetzlar, Germany) at 40× magnification and analysed the percentage of stained tissue using Visiopharm software (Visiopharm, Horsholm, Denmark).

### 2.12 TNBC metastases formation assay

A suspension of 2 x 10^5^ 4T1 Luc‐GFP cells in HBSS was injected into the tail vein of Balb/c nude mice under isoflurane anaesthesia. Bodyweight evolution was followed twice a week. Mice were killed by cervical dislocation after 12 days. Ten minutes before killing, 0.15 mg/g of K + d‐luciferin (Perkin Elmer) was injected intraperitoneally. During killing, lungs were inflated with 1 mL of a 15 mg/mL solution of K + d‐luciferin. Lungs and brain were collected and bathed separately in the K + d‐luciferin solution for 5 minutes. Bioluminescent signals were acquired during 10 minutes with a Xenogen IVIS 50 bioluminescence imaging system (Perking Elmer), and signal quantification was performed with Living Image software (Perkin Elmer).

### 2.13 Statistical analysis

Unpaired *t* test, one‐way ANOVA followed by Turkey's multiple comparison test, two‐way ANOVA followed by Bonferroni's multiple comparison test and Fisher's exact test were performed via GraphPad Prism 7 (GraphPad Software, San Diego, CA, USA), with *P* ≤ .05 considered significant. Results are represented as mean + SEM.

## 3 RESULTS

### 3.1 Association between obesity and TNBC growth in vivo

**FIGURE 1:**
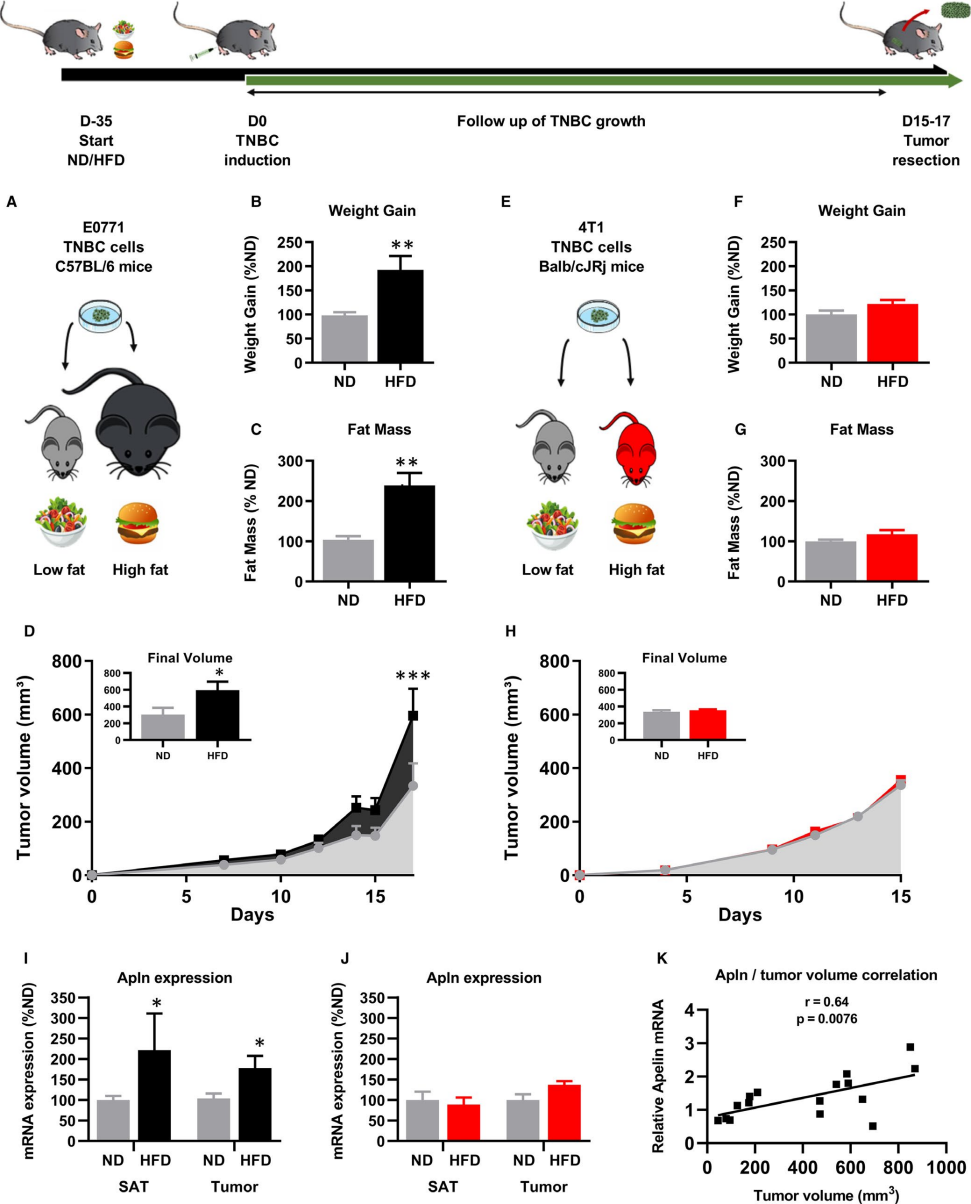
Obesity development favours TNBC growth. A, C57BL6 obesity sensitive model. B, C, Bodyweight gain (B) and total fat mass (C) in C57BL6 models after 5 wk of diet. D, E0771 TNBC growth in ND/HFD‐fed C57BL6 mice. E, Balb/cJRj obesity‐resistant model. F, G, Bodyweight gain (F) and total fat mass (G) in Balb/CJRj mice after 5 wks of diet. H, 4T1 TNBC growth in ND/HFD‐fed Balb/cJRj mice. I, Expression of apelin in subcutaneous adipose tissue (SAT) of C57BL6 mice and E0771 TNBC tumours. J, Expression of apelin in SAT of Balb/cJRj mice and 4T1 TNBC tumours. K, Correlation between E0771 tumour apelin expression and E0771 tumour volume. Data are presented as mean ± SEM. Number of mice per group for (A‐D, I tumour, K): ND: 9, HFD: 5‐6, for (E‐H, J tumour): ND: 5‐6, HFD: 5‐6. Number of subcutaneous adipose tissue per group for I: ND: 8, HFD: 3, for J: ND: 6, HFD: 5. Data were analysed using Student's *t* test for B, C; F, G; I‐L. Data were analysed using two‐way ANOVA followed by Bonferroni post hoc test for D, H. Data were analysed using Pearson's correlation coefficient for K. *P < 0.05; **P < 0.01; ***P < 0.001

To investigate the potential role played by apelin on tumour growth, we combined different models and pharmacological approaches. First, we tested the association between obesity and TNBC growth in vivo. Obesity was induced by feeding C57BL/6 mice with a high‐fat diet (HFD) for 5 weeks (Figure 1A). Compared with mice receiving a normal diet (ND), mice fed a HFD had a significant increase of bodyweight gain and fat mass accumulation (by about 200%) after 5 weeks (Figure 1B,C). Next, we injected E0771 TNBC cells derived from C57BL/6 mice into the mammary fat pad and monitored tumour growth. We observed that development of obesity significantly increased TNBC growth, leading to tumours having twice the volumes of the ND group after 17 days (Figure 1D). In order to discriminate the impact of obesity‐related disorders on TNBC growth from the impact of the HFD itself, we tested the growth of 4T1 TNBC cells derived from Balb/cJRj mice, which are described as an obesity‐resistant model upon HFD feeding (Figure 1E). After 5 weeks of either ND or HFD, Balb/cJRj mice showed no difference of bodyweight gain or fat mass accumulation (Figure 1F,G), confirming resistance to obesity development. We followed 4T1 TNBC growth after orthotopic injection in Balb/cJRj mice and found that HFD alone had no impact on TNBC growth (Figure 1H), thereby showing the necessity to develop obesity and increase fat depots to favour tumour growth. To further explore whether apelin could be associated with these phenotypes, we measured apelin mRNA expression. We found that besides an increased TNBC growth, apelin mRNA expression increased by 120% in the subcutaneous adipose tissue of obese mice (Figure 1I). Interestingly, TNBC that is growing in obese mice also displays increased tumoral apelin expression by approximately 80% compared with TNBC harvested from mice fed a ND (Figure 1I). By contrast, the obesity‐resistant mice showed no difference in apelin expression in the adipose tissue and had a tendency of only ±35% increase tumoral apelin (Figure 1J). Moreover, increased apelin expression in E0771 TNBC correlates with bigger TNBC volumes (Figure 1K), suggesting that increased apelin expression might be one of the mechanisms involved in the increase TNBC growth of obese mice.

### 3.2 Obesity relevant infusion of apelin favours TNBC growth

**FIGURE 2:**
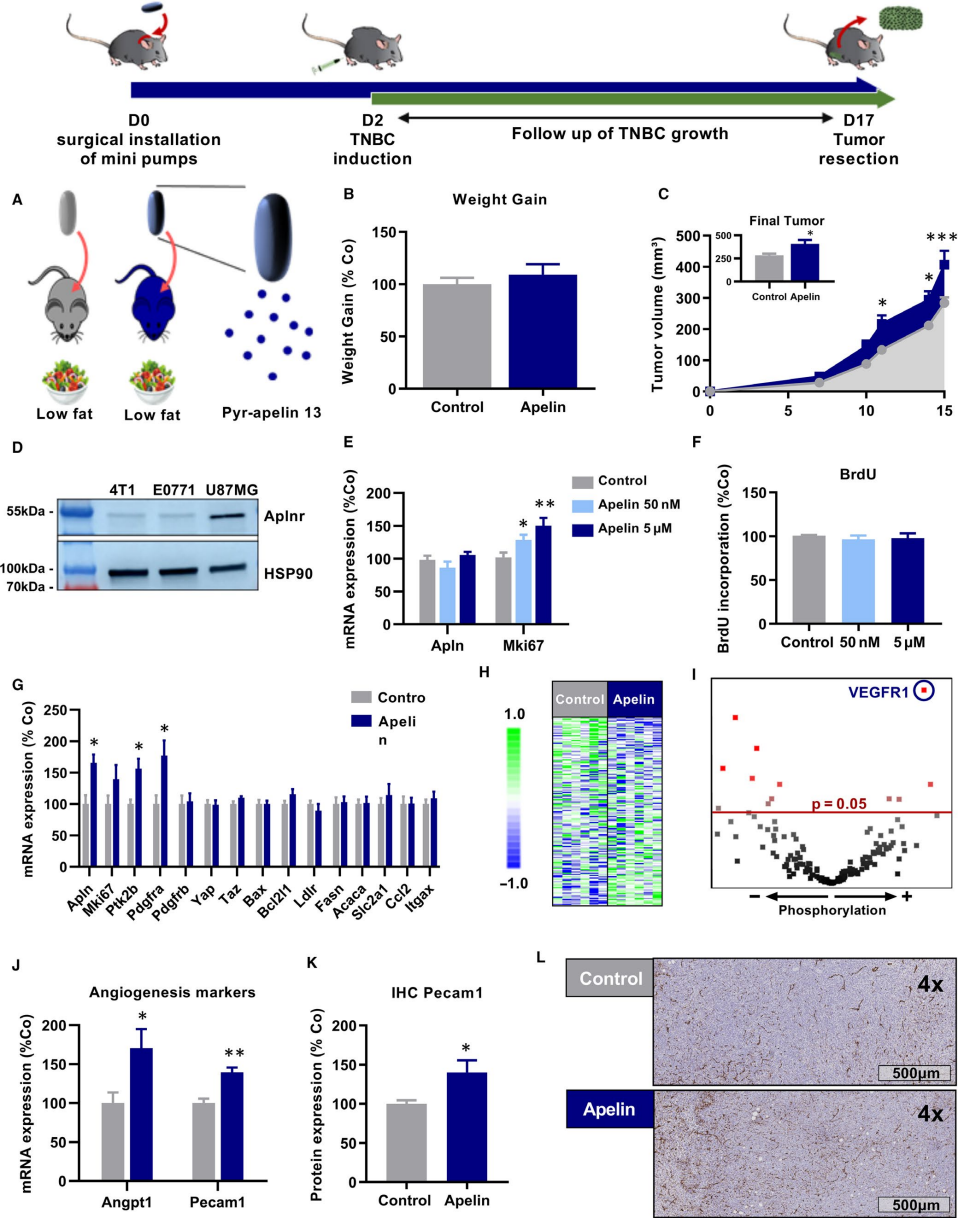
Infusion of obesity‐related levels of apelin favours TNBC growth in lean mice. A, Experimental design of PBS or apelin infusion (0.1 µmol/kg/d) in lean balb/cJRj mice. B, Bodyweight gain during apelin infusion. C, 4T1 TNBC growth in Balb/cJRj mice. D, Expression of Aplnr protein in 4T1 & E0771 TNBC cells with U87MG cells as positive control. E, mRNA expression of Apln and Mki67 in 4T1 TNBC cells in vitro in response to 24 h of apelin treatment. F, BrdU incorporation assay in 4T1 TNBC cells in vitro in response to 24 h of apelin treatment. G, mRNA expression of Apln, tumoral progression markers (Mki67, Ptk2b, Pdgfra, Pdgfrb, Yap, Taz), apoptosis markers (Bax, Bcl2l1), lipid metabolism markers (Ldlr, Fasn, Acaca), glucose metabolism marker (Slc2a1) and immune markers (Ccl2 & Itgax) in 4T1 TNBC tumours. H, I, Tyrosine phosphorylation of 196 peptides from 4T1 TNBC tumours. J, Expression of Angpt1 and Pecam1 in 4T1 tumours. K, L, Immunohistochemistry of Pecam1 on 4T1 TNBC tumours. Data are presented as mean ± SEM. Number of mice per group for (B, C): Control: 15, Apelin: 16, for (E, F): n = 3, for (G‐L) Control: 5‐6, Apelin 5‐6. Data were analysed using Student's *t* test for (B, G, J, K) or using one‐way ANOVA for (E, F). Data were analysed using two‐way ANOVA followed by Bonferroni post hoc test for (C). *P < 0.05; **P < 0.01; ***P < 0.001

To further investigate the potential implication of apelin in the association between obesity and TNBC growth, we reproduced the increase of apelin observed during obesity by chronically administering apelin using osmotic mini pumps. In order to discriminate the influence of apelin on tumour growth from obesity‐related protumoral mechanisms, we performed this experiment in ND‐fed obesity‐resistant Balb/cJRj mice injected with 4T1 TNBC (Figure 2A). Mice infused with apelin for 17 days had no difference in bodyweight gain compared with PBS‐infused mice (Figure 2B). Interestingly, in two independent experiments, the infusion of apelin facilitated 4T1 TNBC growth in lean mice, leading to significantly bigger tumour sizes (Figure 2C) than in vehicle‐treated animals. In order to understand the mechanisms involved in the increased 4T1 TNBC growth upon apelin infusion, we tested a potential effect of apelin treatments on in vitro proliferation of 4T1 TNBC cells. Western blot analysis revealed low basal expression of Aplnr in 4T1 TNBC cells (Figure 2D). Despite this, treatment of 4T1 cells with apelin significantly increased mRNA expression of the proliferation marker Mki67 that has been previously correlated with poor prognosis (^44^; Figure 2E). However, the increased Mki67 expression did not favour proliferation of 4T1 cells as such in our experimental conditions (Figure 2F). This suggests that Aplnr expression in 4T1 TNBC cells might be too low to directly affect cell proliferation. Ex vivo analysis of infused tumours revealed that apelin infusion significantly increased tumour apelin mRNA expression (Figure 2G). However, apelin infusion had a limited impact on the expression of different key genes involved in cancer cells proliferation, survival and energy metabolism (Figure 2G). These results further supported the hypothesis of a lack of direct Aplnr activation in 4T1 TNBC cells. Therefore, we hypothesized that apelin protumoral effects might come from a modulation of the tumour microenvironment. Hence, we performed a tyrosine kinase activity assay (*PamGene*) on tumours samples to analyse the signalling pathways that were impacted by apelin. Globally, apelin had a subtle but clear impact on tyrosine phosphorylation profile (Figure 2H,I). We found that apelin infusion increases tyrosine phosphorylation of the key angiogenic marker vascular endothelial growth factor receptor 1 (VEGFR1) (Figure 2I), thereby suggesting an increased tumour neoangiogenesis. This hypothesis was further confirmed by the significant increase of angiopoietin 1 and Pecam1 mRNA expression (Figure 2J) and by histological analysis of Pecam1 staining in apelin‐infused tumours (Figure 2K,L).

### 3.3 Obesity relevant infusion of apelin favours TNBC brain metastases

**FIGURE 3:**
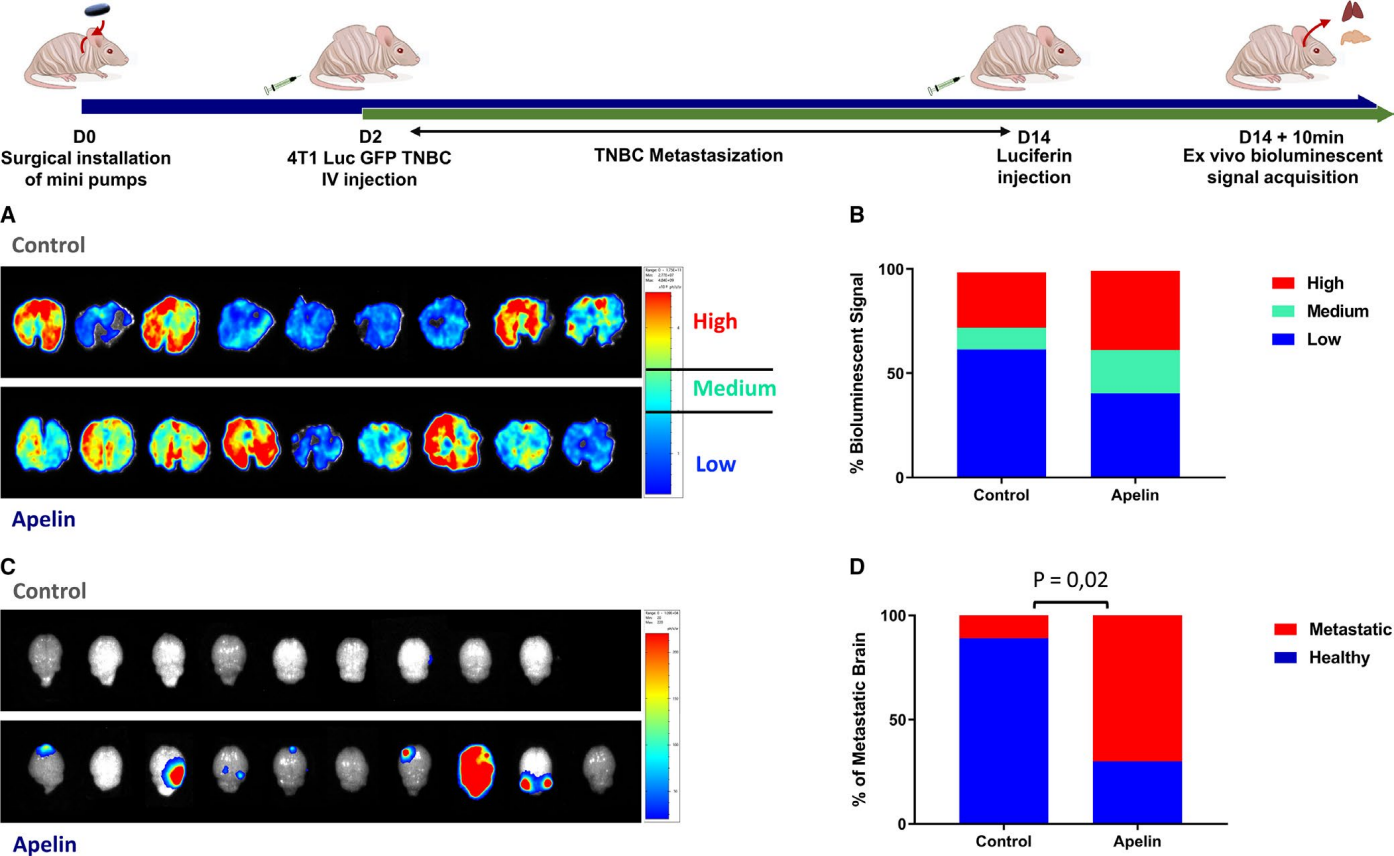
Infusion of obesity‐related levels of apelin favours TNBC brain metastatization. A, Ex vivo bioluminescent signals from 4T1 Luc‐GFP in lungs of PBS or apelin‐infused (0.1 µmol/kg/d) Balb/c nude mice. B, Quantification of bioluminescent signals of lung metastases by intensity. C, Ex vivo bioluminescent signals from 4T1 Luc‐GFP in brains of PBS or Apelin‐infused Balb/c nude mice. D, Quantification of metastatic brains in PBS or Apelin‐infused mice. Number of mice per group for (A, B): Control: 9, Apelin: 9, and for (C, D): Control: 9, Apelin: 10. Data were analysed using two‐way ANOVA followed by Bonferroni post hoc test for (B). Data were analysed using Fisher's exact test for (D)

We next tested whether obesity‐related levels of apelin could also influence TNBC metastasization in addition to an increase in primary TNBC tumour growth. In order to detect metastases efficiently, we injected 4T1 TNBC cells harbouring a fluorescent Luc‐GFP transgene into the tail veins of ND‐fed Balb/c nude mice with or without infusion of apelin. Breast cancer cells are known to form metastases in diverse distant tissue like the lungs and brains. Indeed, after a period of 12 days of metastasization, the lungs of both PBS and apelin‐infused mice were metastatic (Figure 3A). However, mice infused with apelin tended to have more highly metastatic areas (Figure 3B). Interestingly, 70% of apelin‐infused mice had metastases in brain whereas most of the brains of the PBS‐infused group were metastases‐free (Figure 3C,D). This set of data highlights the significant prometastatic effect of apelin.

### 3.4 Apelin as a therapeutic target for TNBC growth in obese mice

**FIGURE 4:**
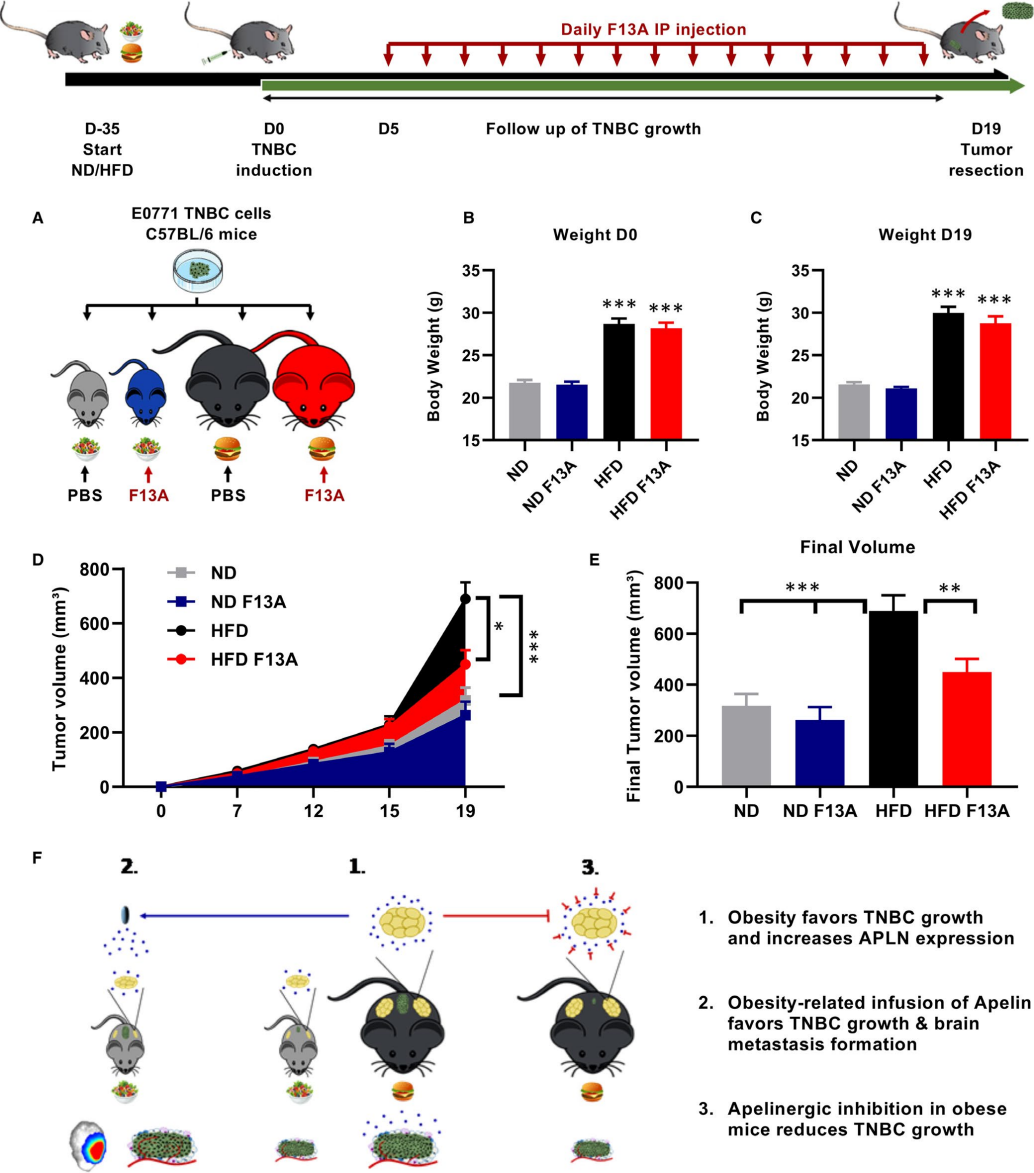
Apelinergic antagonist abolishes the impact of obesity on TNBC growth. A, Experimental design of daily PBS or F13A (0.5 µg/g) injections in lean or obese C57BL6 mice. B, Bodyweight after 5 wk of ND/HFD. C, Bodyweight at the end of tumour growth and treatment. D, E0771 TNBC growth in lean or obese C57BL6 mice injected with either PBS or F13A. E, Final E0771 TNBC volumes. Number of mice per group for (A‐F): ND: 16, ND F13A: 15, HFD: 20, HFD F13A: 22. Data were analysed using one‐way ANOVA followed by Bonferroni post hoc test for (B, C, E). Data for (D) were analysed with two‐way ANOVA followed by Bonferroni post hoc test. *P < 0.05; **P < 0.01; ***P < 0.001

Having established that the administration of apelin to lean mice, thereby mimicking the obesity situation, led to an increased TNBC growth and brain metastases formation, we next wondered whether the apelinergic system could be a realistic therapeutic target for TNBC in obese conditions. To test this, we administrated a commonly used apelinergic antagonist, F13A, during TNBC growth in lean or obese C57BL6 mice (Figure 4A). Similar to the first experiment, we fed C57BL6 mice either a ND or HFD for 5 weeks in order to trigger development of obesity (Figure 4B). After 5 weeks of diet, we injected E0771 TNBC cells in the mammary fat pad of lean and obese mice. After 5 days of tumour growth, we started daily intraperitoneal injections of either PBS or F13A. We conducted two independent experiments with these parameters. First, we observed that F13A injections did not affect the obese phenotype as the two HFD‐fed groups had the same bodyweight at the end of the experiment (Figure 4C). Second, we confirmed that obesity development with HFD favoured TNBC growth compared with the ND group (Figure 4D). Interestingly, we found that F13A injections in obese mice significantly reduced TNBC growth compared with PBS‐injected obese mice (Figure 4D) leading to significantly lower final tumour volume (Figure 4E). These results suggest that interfering with the apelinergic system in obesity could be an interesting therapeutic strategy (Figure 4F).

## 4 DISCUSSION

During the past few years, several studies have shown a correlation between tumour growth and plasma levels or tumoral expression levels of apelin.^38^, ^39^, ^40^. Moreover, the artificial modulation of apelin expression in cancer cell lines by knockout (^45^) or induced overexpression^41^ has suggested a direct implication of apelin on cancer progression. Although informative, these experiments remain descriptive and artificial because they are focusing only on apelin expression in cancer cells. Hence, the implication of the host circulating apelin on tumour progression remains largely unstudied. The main condition known to modulate circulating apelin levels is the development of obesity that increases apelin expression in adipose tissue.^31^

In this study, we tested the impact of obesity and HFD alone on TNBC growth. Only the development of obesity led to an increased tumour growth, suggesting that a diet rich in lipids is not sufficient to promote TNBC progression and that obesity conditions are required. One contributing factor to tumour growth in obesity could be the modulation of apelin expression as it is increased in obese mice showing large TNBC tumours but unaffected in HFD‐fed mice showing normal TNBC tumours. We confirmed a causal link between increased apelin expression in obese mice and larger TNBC tumours with two different approaches. First, by accurately mimicking apelin levels as observed in obesity condition, we showed that apelin is sufficient to promote TNBC growth in lean mice and to increase tumour angiogenesis. This effect is independent of obesity development as apelin infusion did not affect the bodyweight. Next, we showed that antagonizing the Aplnr in obese mice limits TNBC growth. This reduction of TNBC growth occurs without the necessity of targeting obesity confirming a key implication of apelin protumoral effects in the context of obesity.

It has been estimated that roughly 70% of breast cancer deaths are caused by metastases (^46^). In our experiments, the reproduction of obesity‐related levels of apelin in lean mice led to a marked increase of brain TNBC metastase production. A trend to form more highly metastatic area in the lungs was also observed. As TNBC cells in mice form brain metastases only after lung metastases, it might suggest that the tumoral spread was already too advanced in our experimental design to correctly assess secondary tumour formation in the lungs. Interestingly, we did not observe brain metastases in the control group, indicating that apelin facilitated the seeding of circulating tumours cells into the brain. This would imply that obese patients suffering from TNBC have a potential additional risk of being more prone to develop secondary tumours in the brain.

Several studies in human beings have shown a correlation between high Aplnr expression in cancer and increased tumour invasion or overall poorer prognosis (^47^; ^48^). Our experiments were performed with TNBC cell lines with low expression of Aplnr (Figure 2D) and our in vitro data showed that apelin treatment did not have a direct impact on 4T1 TNBC cell proliferation. However, we could have missed a potential proliferative effect of apelin by not expanding treatment beyond 24 hours as apelin treatment increased Mki67 mRNA expression. Despite the limited expression of Aplnr and a suggested lack of proliferative effect in 4T1 TNBC cells, the reproduction of obesity‐related levels of apelin was able to promote 4T1 TNBC growth and metastasization. Moreover, the use the antagonist F13A in our experiment reduced the growth of TBNC regardless of low Aplnr expression in E0771 TNBC cells. This suggests that apelin drives tumour growth mainly by acting on the tumour microenvironment. We can hypothesize that apelin could have a greater impact on tumour progression by activating proliferation of cancer cells with high Aplnr expression as suggested by one in vitro study performed with the MCF7 BC cell line (^49^) with a high Aplnr expression. However, our results show that targeting the apelinergic system is sufficient to reduce tumour growth and therefore suggest that this can be achieved without directly targeting Aplnr in TNBC.

A correlation between high circulating apelin and tumour grade or overall bad prognosis has previously been described.^38^ On the other hand, another study suggested that tumour apelin expression was more accurately correlated with increased tumour progression.^39^ In our work, we showed that in obese mice or in mice in which apelin was infused to attain obesity‐like levels, TNBC growth was facilitated, but also that tumour apelin expression was increased. Different mechanisms are possibly involved. First, high circulating apelin may not affect tumour growth directly but rather increase tumour apelin expression. In this scenario, tumour apelin would be the main driver of TNBC progression and circulating apelin would only have an indirect effect on TNBC growth by increasing tumour apelin expression. Second, high circulating apelin could promote tumour growth but also increase tumour apelin expression directly, generating a positive feedback loop that drives TNBC progression. In this case, both the circulating and the subsequent local apelin expression would favour TNBC growth in a direct manner. Third, high circulating apelin could promote tumour growth without affecting tumour apelin expression. The increased tumour apelin expression could be a characteristic of highly proliferative cancer cells in more advanced tumours rather than a direct consequence of high circulating apelin. In this scenario, high circulating apelin levels promote TNBC growth and the subsequent fast‐growing tumours would increase their apelin expression in order to promote angiogenesis and support their growth. A fourth mechanism, quite similar to the previous, could be that as high circulating apelin promote tumour neoangiogenesis, the apelin expression measured in the tumour could originate from proliferating endothelial cells rather than cancer cells.

In this study, we deliberately dissociated increased apelin levels caused by obesity from additional disorders associated with obesity and found that the increased TNBC growth is slightly smaller than the effect observed in obese mice. An explanation probably lies in the fact that obese mice have obesity‐linked disorders that can further promote tumour growth, such as increase in hyperinsulinaemia or high leptin expression.^17^, ^26^ This hypothesis is supported by the fact that the apelinergic antagonist F13A in obese mice did not completely abolish the impact of obesity on TNBC growth. Another possibility could be that the apelin infusion used in this study cannot account for all effects on the apelinergic system in obesity conditions. Indeed, a second ligand of Aplnr has been recently discovered, a peptide called ELABELA (Toddler/Apela) (^50^). To our knowledge, there is not measurement of ELABELA levels in obese subjects or mice. Taking into account, the obesity‐related levels of ELABELA and apelin for an infusion in lean mice could reproduce even more accurately the activation of the apelinergic system in obesity. Such an infusion could have an even greater impact on TNBC growth than the already significant increase described in this study.

Finally, apelin is also an adipokine that has recently been proposed as a key target to treat type 2 diabetes^37^ or sarcopenia (^51^) in human beings. Indeed, apelin has gained attention for its ability to increase insulin sensitivity and its beneficial role on glucose homeostasis. In line with these effects, apelin is now considered as a potential therapeutic agent for diabetic patients. However, many patients suffering from type 2 diabetes have a BMI corresponding to obesity. Hence, extreme caution would be required to select patients who could benefit from apelin as a therapeutic agent because our results strongly suggest that either obesity‐related levels of apelin as well as apelin infusion is able to increase TNBC growth and seeding to peripheric tissues, like the brain. Therefore, the balance risk–benefit effects of using apelin as a therapeutic target warrant careful assessment.

In this study, we showed for the first time that obesity‐increased apelin expression favours breast cancer progression. We suggest that apelin is a critical driver of obesity‐induced TNBC progression. We found that blocking the Aplnr during obesity reduces tumour growth likely by affecting tumour microenvironment thereby highlighting that apelinergic system interference could be an interesting therapeutic strategy in the context of obesity and TNBC.

## CONFLICTS OF INTEREST

The authors declare no conflict of interest.

## AUTHOR CONTRIBUTION

**Florian Gourgue:** Conceptualization (equal); Data curation (equal); Formal analysis (equal); Investigation (equal); Methodology (equal); Resources (equal); Validation (equal); Writing‐original draft (equal). **Lionel Mignion:** Data curation (equal); Investigation (equal); Methodology (equal); Resources (equal); Writing‐review & editing (equal). **Matthias Van Hul:** Methodology (equal); Resources (equal); Writing‐review & editing (equal). **Natacha Dehaen:** Investigation (equal). **Estelle Bastien:** Data curation (equal); Formal analysis (equal); Investigation (equal); Methodology (equal); Resources (equal). **Valery Payen:** Investigation (equal); Methodology (equal); Resources (equal). **Baptiste Leroy:** Investigation (equal); Methodology (equal); Resources (equal). **Nicolas Joudiou:** Investigation (equal); Methodology (equal); Resources (equal). **Didier Vertommen:** Investigation (equal); Methodology (equal). **Caroline Bouzin:** Investigation (equal); Methodology (equal); Resources (equal); Writing‐review & editing (equal). **Nathalie Delzenne:** Resources (equal). **Bernard Gallez:** Resources (equal). **Olivier Feron:** Resources (equal); Writing‐review & editing (equal). **Benedicte F Jordan:** Conceptualization (equal); Funding acquisition (equal); Supervision (equal); Validation (equal); Writing‐review & editing (equal). **Patrice D Cani:** Conceptualization (equal); Funding acquisition (equal); Supervision (equal); Validation (equal); Writing‐review & editing (equal).

## Data Availability

The data that support the findings of this study are available from the corresponding authors upon request.
